# Relationship between Trace Elements and Matrix Metalloproteinases 2 and 9 and their Tissue Inhibitors in Medullary Thyroid Carcinoma

**DOI:** 10.1007/s12011-022-03431-z

**Published:** 2022-09-26

**Authors:** Héctor Vázquez-Lorente, Duško M. Dundjerović, Svetislav B. Tatić, Sara Rodríguez-Menéndez, Héctor González-Iglesias, Cláudio M. Gomes, Ivan R. Paunović, Vesna V. Dragutinović

**Affiliations:** 1 Department of Physiology, Faculty of Pharmacy, University of Granada, Granada, Spain; 2 Institute of Pathology, Faculty of Medicine, University of Belgrade, Belgrade, Serbia; 3 Instituto Universitario Fernández-Vega (Fundación de Investigación Oftalmológica, Universidad de Oviedo), Oviedo, Spain; 4 Instituto de Productos Lácteos de Asturias, Consejo Superior de Investigaciones Científicas (IPLA-CSIC), Villaviciosa, Spain; 5 Biosystems & Integrative Sciences Institute, Faculdade de Ciências, Universidade de Lisboa, Lisbon, Portugal; 6 Departamento de Química E Bioquímica, Faculdade de Ciências, Universidade de Lisboa, Lisbon, Portugal; 7 Faculty of Medicine, University of Belgrade, Belgrade, Serbia; 8 Center for Endocrine Surgery, Clinical Centre of Serbia, Belgrade, Serbia; 9 Institute of Chemistry in Medicine, Faculty of Medicine, University of Belgrade, Belgrade, Serbia

**Keywords:** Medullary thyroid carcinoma, Trace elements, Calcitonin, Matrix metalloproteinases (MMPs), Tissue inhibitors of matrix metalloproteinases (TIMPs)

## Abstract

Medullary Thyroid Carcinoma (MTC) constitutes around 5% of all thyroid cancers. Trace elements assessment has emerged as a useful strategy in the diagnostics of MTC combined with Matrix Metalloproteinases (MMPs) and Tissue Inhibitors of Matrix Metalloproteinases (TIMPs) analysis. The aim of this study was to compare the presence and content of trace elements (i.e., Copper (Cu), Zinc (Zn), Iron (Fe), and Manganese (Mn)) in MTC with respect to control samples and their potential relationship with markers of MTC in tissues. The study included 26 patients who had undergone thyroidectomy, due to the diagnosis of MTC and 17 patients as control. We combined tumour pathology and staging, immunohistochemical analysis of calcitonin, MMPs, and TIMPs, with analytical biochemistry using Inductively Coupled Plasma – Mass Spectrometry (ICP-MS) to determine the levels of trace elements. No differences by MTC type for MMPs and their TIPMs, although strong TIMP-1 and TIMP-2 immunohistochemical expression of MTC were unveiled. Additionally, Zn, Fe, and Mn tended to be decreased, and Cu to be increased in samples presenting MTC with respect to controls. Moreover, Zn was the unique trace element which seemed to be correlated with MMPs and TIMPs. Trace elements such as Zn, Fe, and Mn are decreased in tissues affected by MTC. In addition, Zn may be the trace element which saves more relationship with the proportion and intensity of MMPs, being considered altogether useful biomarkers of MTC. We therefore suggest the analysis of novel and traditional markers of MTC as a novel approach in this pathology.

**Supplementary Information:**

The online version contains supplementary material available at 10.1007/s12011-022-03431-z.

## Introduction

Medullary Thyroid Carcinoma (MTC) is a form of thyroid carcinoma originated in the parafollicular cells, where calcitonin hormone – a sensitive biomarker used for its diagnosis – is produced. MTC, which can be both sporadic and hereditary, constitutes around 5% of all thyroid cancers, being in the third place of most frequent thyroid carcinomas [1–5].

MTC is characterized by the presence of high levels of calcitonin and the Zinc (Zn) peptidase–Matrix Metalloproteinases 2 and 9 (MMP-2 and MMP-9) and their Tissue Inhibitors of Matrix Metalloproteinases (TIMPs) [6, 7]. MMPs are overexpressed in a variety of malignant tumours and are a unique family of Zn-binding endopeptidases that can degrade the components of the extracellular matrix, which is essential in invasive growth and metastasizing processes [8]. In this regard, MMPs have a different role in tumour progression depending on the enzymatic activity, the type of enzyme producing cells, and the tumour stages [9]. Additionally, expression of TIMPs, which are proxies of tumour progression, are associated with angiogenesis, invasion, and metastasis in carcinogenic processes [10].

Trace elements assessment has emerged as a useful strategy in the diagnostics of MTC combined with MMPs and their TIMPs analysis. Zn plays a substantial role in (i) cell proliferation, (ii) the regulation of chronic inflammatory status reducing inflammatory cytokines, (iii) the reduction of oxidative stress via synthesizing antioxidant enzymes, and (iv) catalysing multiple enzymes [11]. A recent study suggested that the status of certain trace elements such as Zn are important in MTC pathophysiology – their levels being increased and a potent risk factor for this type of carcinoma – [12]. The relationship between Zn and thyroid metabolism is based on the hypothesis that the nuclear T3 receptor contains Zn binding protein [13].

However, although the role of Zn upon MTC has been widely studied, the role of other trace elements (e.g., Copper (Cu), Iron (Fe) and Manganese (Mn)) on MTC has not been previously elucidated or has been studied in other types of thyroid carcinomas (i.e., papillary thyroid carcinoma (PTC)) [14]. Therefore, the aim of this study was to compare the presence and content of trace elements (i.e., Cu, Zn, Fe, and Mn) in MTC with respect to control samples and their potential relationship with markers of MTC in tissues.

## Materials and Methods

### Study Participants

The present study included 26 MTC samples (73% women) aged 21 to 77 years who had undergone thyroidectomy at the Centre for Endocrine Surgery of the Institute of Endocrinology, Diabetes and Metabolic Diseases, Clinical Centre of Serbia, Belgrade, during 2012 and 2013. Seventeen samples were included in the study as controls – their tissue samples being obtained from patients treated for benign nodular hyperplasia –.

This study abides by the Declaration of Helsinki on research involving human subjects and was approved by the Ethics Committee of the Faculty of Medicine, University of Belgrade No. 29/XI-10 for studies involving human subjects. The samples’ identity remained anonymous. Written informed consent was obtained from all individual participants after explaining goals of the study.

Classification according to International Union Against Cancer was applied to classify tumours. Groups were formed by the following criteria: T category 1 (≤ 20 mm) and T category 2 (20–40 mm) [15].

### Samples Treatment and Measurement

#### Calcitonin in Serum Analysis

Calcitonin in serum was determined by chemiluminescent immunoassays on the automatic Analyzer IMMULITE 100 Siemens (Germany).

#### Tissue Microarrays (TMA)

Samples were fixed with 10% formalin and embedded in paraffin blocks. A high-density TMA was constructed manually. Previously marked area of interest on slides was translated to corresponding regions of donor paraffin blocks. Needle with inner diameter of 1.1 mm was used to create and transfer tissue cores (0.785 mm^2^ crosscut surface area) in recipient paraffin blocks. Two cores were taken from every lesion. Cases with at least one section across all slides were regarded as valid. Tissue cores with external controls were included in all TMAs. Final TMA block consisted of 60 cores (10 × 6), plus two control tissue cores. Control tissues included in TMA were normal thyroid tissues [16–19].

#### Immunohistochemistry

Immunohistochemical staining of Calcitonin in Tissues (CT) (DAKO, pAb, RTU), MMP-9 (Sigma-Aldrich, pAb, 1:300), MMP-2 (Sigma-Aldrich, pAb, 1:50), TIMP-1 (Abcam, Clone 102D1, 1:100), and TIMP-2 (Abcam, Clone 3A4, 1:100) was done manually according to the manufacturer instructions as shown in Supplementary Table 1.

#### Evaluation of Immunohistochemical Staining

Cytoplasmatic ± membranous immunoreactivity for CT, MMP-9, MMP-2, TIMP-1, and TIMP-2 in more than 10% of cells was considered as positive staining without regard to intensity of staining. In respect to percentage of positive cells (P) of staining, we graded staining as 0, 1, 2, 3, when 0–10%, 11–25%, 26–50%, 51–100% of tumours cells showed expression, respectively. We also scored the overall intensity (I) such as 0, 1, 2, 3 for no staining at all, weak, moderate, and intensive staining, respectively. All the previous immunohistochemical procedures were followed according to standard protocols [20].

#### Trace Elements Analysis

A quadrupole Inductively Coupled Plasma – Mass Spectrometry (ICP-MS) system (model Agilent 7500ce from Agilent Technologies, Tokyo, Japan) was used for total multi-element quantification. Torch position and ion lens voltage settings of ICP-MS were optimized daily for optimum sensitivity and control of oxide formation with a multi-element standard solution (1 ng/mL^−1^ Li, Co, Y, Ce and Tl, in 1% w/w HNO_3_). The following trace elements were monitored by ICP-MS 7500ce: Fe, Mn, Cu, and Zn. The quadrupole ICP-MS was equipped with an octupole collision cell to remove polyatomic interferences for selected analytes, operating the collision cell with a H_2_ gas flow of 4.0 mL·min^−1^. Optimized operating conditions are shown in Supplementary Table 2.

#### Quantitative Multi-element Analysis of Zn, Cu, Fe and Mn in Paraffin Embedded MTC and Control Samples

Total quantification of essential trace elements (i.e., Zn, Fe, Cu and Mn) was carried out by conventional nebulization ICP-MS after acidic digestion of the 26 paraffin-embedded tissues which were mineralized. Briefly, samples weighing from 10 to 30 mg were digested using 750 µL of concentrated high purity grade HNO_3_ (65% v/v, Suprapur® Merck, Germany) and heated 2 h at 90 °C, under high pressure. Preparatory blank tubes containing paraffin (no tissue added) were similarly treated as samples and used as background controls. Mineralized solutions were diluted with Milli-Q water to reach 2% of HNO_3_, using Ga (10 ng·mL^−1^) as internal standard. External calibration curve of Zn, Cu, Fe, and Mn were performed by the preparation of Zn, Cu, Fe and Mn standard solutions (0–250 ng·mL^−1^) in 2% HNO_3_ using Ga (10 ng·mL^−1^) as internal standard. Control samples were prepared following the same procedure.

### Statistical Analysis

As a previous step to the execution of a parametric model or not, the hypothesis of normal distribution was rejected using the Shapiro–Wilk test, visual check of histograms, Q-Q, and box plots. Descriptive statistics have been used for data expression of age, calcitonin in serum, Cu, Zn, Fe, and Mn, indicating the results of the numerical variables such as Median (M) and Interquartile Range (IQR). Frequency statistics was used for data expression of category of tumour, sex, MMPs, TIMPs, and CT, indicating the results of the variables as frequency (N) and percentage (%). Mann–Whitney test was used to compare the median distribution of calcitonin in serum and trace elements by median age. Chi square test was used for correlating categorical variables, i.e., the relationship between immunohistochemistry of MTC markers and the category of tumour. Linear correlation analysis was used to estimate the degree of association between trace elements and MMPs and their TIMPs using Spearman’s correlation coefficient (Rho). Statistical significance was defined as *p* value < 0.05. The statistical analysis was performed using the SPSS 22.0 statistical program for MAC (SPSS Inc. Chicago, IL, USA). For the graphical plots, GraphPad Prism 8 software (GraphPad Software, San Diego, CA, USA) was used.

## Results

The general characteristics of the samples of this study are represented in Table 1. One-third of the samples presented T2 MTC. Moreover, females represented more than two-thirds of the samples with MTC. Calcitonin in serum, Cu, Zn, Fe, and Mn, showed no median differences by age (all *P* > 0.05).

**Table 1:** General characteristics of the samples

	*N*	*N* T1 (%)	*N* T2 (%)
T category of tumour	26	10 (38)	16 (62)
	*N*	*N* Males (%)	*N* Females (%)
Sex	26	7 (27)	19 (73)
	*N*	Median	IQR
Age (years)	26	53.0	22.8
Calcitonin in serum (ng/L)	26	582.5*	1365.4
Copper (µg/g)	26	3.96*	3.25
Zinc (µg/g)	26	9.60*	11.1
Iron (µg/g)	26	25.5*	32.6
Manganese (µg/g)	26	0.17*	0.18

*N* = 26; Frequency statistics has been used for data expression of T category of tumour and sex indicating the results of the variables as frequency (N) and percentage (%). Descriptive statistics has been used for data expression of age, calcitonin in serum, and trace elements, indicating the results of the numerical variables as Median (M) and Interquartile Range (IQR). Mann–Whitney test was used to compare the median distribution of calcitonin in serum and trace elements by median age. *Non-significance was set at *P*-values ≥ 0.05. Abbreviations: *T1*, T category 1; *T2*, T category 2

**Fig. 1:**
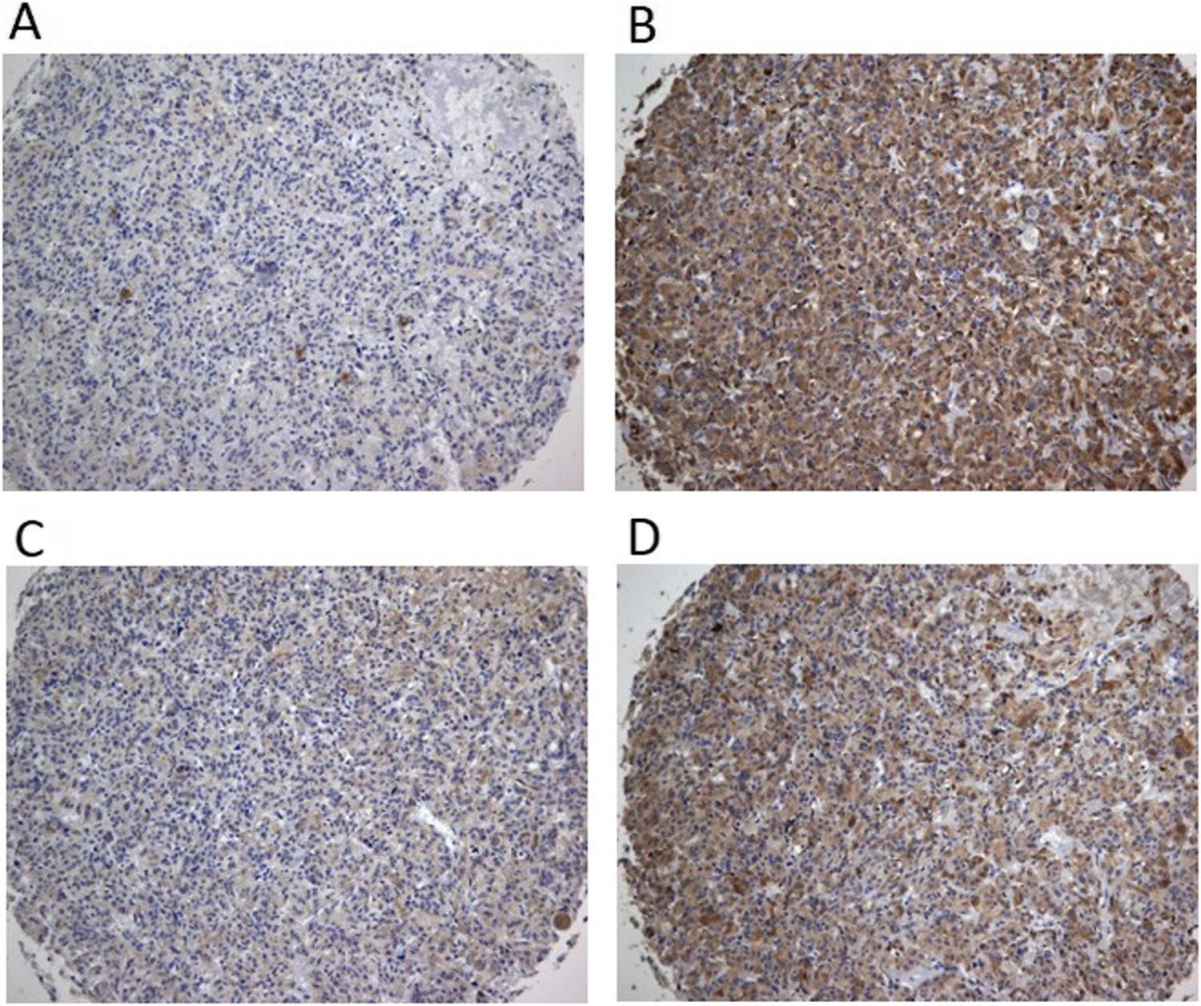
Immunohistochemical expression of matrix metalloproteinases and their tissue inhibitors in medullary thyroid carcinoma. **a** Matrix Metalloproteinase-2, **b** Tissue Inhibitor of Matrix Metalloproteinase-2, **c** Matrix Metalloproteinase-9 and **d** Tissue Inhibitor of Matrix Metalloproteinase-1

The immunohistochemical expression of MMPs and their TIMPs are represented in Fig. 1. The results of our study showed weak-moderate expression MMP-2 and MMP-9 but strong TIMP-1 and TIMP-2 immunohistochemical expression of MTC.

Immunohistochemistry of MTC markers by T category, is found in Table 2. No significant changes were observed for the analyzed parameters by T category of MTC (all *P* ≥ 0.088).

**Table 2:** Immunohistochemistry of the MTC markers of the study

T1		P (*N* (%))				T2	P (*N* (%))				
	N	0	1	2	3	N	0	1	2	3	*P*-value
MMP-2	10	1 (10.0)	4 (40.0)	3 (30.0)	2 (20.0)	16	1 (6.30)	3 (18.7)	8 (50.0)	4 (25.0)	0.616
MMP-9	10	3 (30.0)	3 (30.0)	3 (30.0)	1 (10.0)	16	5 (31.3)	4 (25.0)	4 (25.0)	3 (18.7)	0.935
TIMP-1	10	0 (0.00)	0 (0.00)	1 (10.0)	9 (90.0)	16	0 (0.00)	1 (6.30)	4 (25.0)	11 (68.7)	0.426
TIMP-2	10	0 (0.00)	0 (0.00)	5 (50.0)	5 (50.0)	16	3 (18.7)	4 (25.0)	3 (18.7)	6 (37.4)	0.088
CT	10	1 (10.0)	2 (20.0)	4 (40.0)	3 (30.0)	16	1 (6.30)	3 (18.7)	5 (31.3)	7 (43.7)	0.906
T1		I (*N* (%))				T2	I (*N* (%))				*P*-value
	N	0	1	2	3	N	0	1	2	3	
MMP-2	10	1 (10.0)	4 (40.0)	3 (30.0)	2 (20.0)	16	1 (6.30)	4 (25.0)	4 (25.0)	7 (43.7)	0.654
MMP-9	10	3 (30.0)	2 (20.0)	2 (20.0)	3 (30.0)	16	5 (31.3)	1 (6.70)	5 (31.3)	5 (31.3)	0.728
TIMP-1	10	0 (0.00)	0 (0.00)	4 (40.0)	6 (60.0)	16	0 (0.00)	3 (18.7)	3 (18.7)	10 (63.0)	0.233
TIMP-2	10	0 (0.00)	2 (20.0)	4 (40.0)	4 (40.0)	16	3 (18.7)	1 (6.30)	9 (56.7)	3 (18.7)	0.237
CT	10	1 (10.0)	2 (20.0)	3 (30.0)	4 (40.0)	16	1 (6.30)	5 (31.3)	5 (31.3)	5 (31.3)	0.910

*N* = 26; Frequencies of different stages of P and I of biomarkers by MTC groups were compared using Chi-square test. Statistical significance was defined as *P*-value < 0.05. Abbreviations: *T1*, T category 1; *T2*, T category 2; *P*, Percentage of positive cells; *I*, Intensity; *MMP-2*, Matrix Metalloproteinase-2; *MMP-9*, Matrix Metalloproteinase-9; *TIMP-1*, Tissue Inhibitor of Matrix Metalloproteinase-1; *TIMP-2*, Tissue Inhibitor of Matrix Metalloproteinase-2; *CT*, Calcitonin in Tissue

**Fig. 2:**
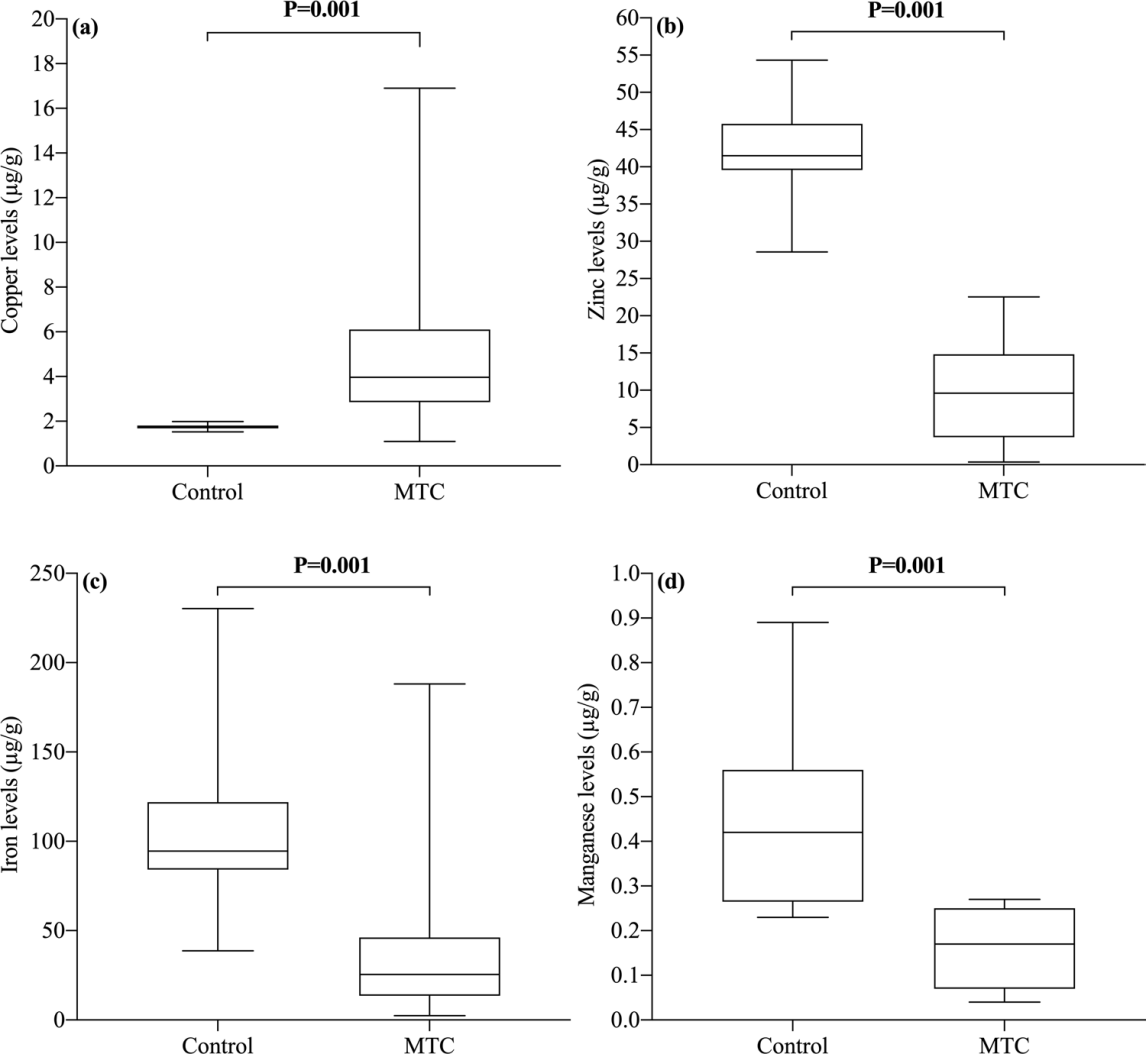
Median differences of trace elements values in MTC group with respect to control group. *N* = 26. For inter-groups analysis, Mann–Whitney test was used. Significance was set at *P*-values less than 0.05. Significant median differences are boldface. Abbreviations: MTC = Medullary Thyroid Carcinoma

Figure 2 shows the median differences of trace elements values in MTC group vs control group. Cu was increased in MTC group with respect to control group (*P* ≤ 0.001; Panel A), whereas Zn, Fe and Mn were decreased in MTC group when compared to control group (all *P* ≤ 0.001; Panels B–D).

Table 3 shows the Spearman’s correlation analysis between trace elements and MMPs, their TIMPs and CT. Zn correlated inversely with I-MMP-2, P-MMP-9, I-MMP-9, and P-CT (all *P* ≤ 0.048), whereas no relationship was observed with the rest of MMPs, their TIMPs and CT (All *P* ≥ 0.119). Cu, Fe, and Mn showed no relationship with MMPs, their TIMPs and CT (all *P* ≥ 0.059).

**Table 3:** Matrix for Spearman’s correlation coefficients (Rho) showing the simple linear relationships between trace elements and MMPs, their TIMPs and CT

	Copper (µg/g)	Zinc (µg/g)	Iron (µg/g)	Manganese (µg/g)
P-MMP-2	Rho = − 0.194 P = 0.344	Rho = 0.093 P = 0.653	Rho = 0.193 P = 0.345	Rho = − 0.219 P = 0.518
I-MMP-2	Rho = 0.293 P = 0.147	**Rho = − 0.447** **P = 0.022**	Rho = − 0.143 P = 0.486	Rho = 0.229 P = 0.260
P-MMP-9	Rho = 0.199 P = 0.331	**Rho = − 0.405** **P = 0.040**	Rho = − 0.090 P = 0.663	Rho = − 0.303 P = 0.365
I-MMP-9	Rho = 0.002 P = 0.993	**Rho = − 0.391** **P = 0.048**	Rho = − 0.129 P = 0.531	Rho = − 0.244 P = 0.470
P-TIMP-1	Rho = 0.150 P = 0.464	Rho = 0.313 P = 0.119	Rho = − 0.070 P = 0.734	Rho = 0.584 P = 0.059
I-TIMP-1	Rho = 0.152 P = 0.458	Rho = − 0.085 P = 0.678	Rho = 0.270 P = 0.182	Rho = 0.452 P = 0.163
P-TIMP-2	Rho = 0.019 P = 0.926	Rho = 0.171 P = 0.403	Rho = 0.121 P = 0.556	Rho = 0.197 P = 0.562
I-TIMP-2	Rho = − 0.057 P = 0.781	Rho = − 0.232 P = 0.254	Rho = − 0.280 P = 0.166	Rho = − 0.060 P = 0.861
P-CT	Rho = − 0.047 P = 0.147	**Rho = − 0.418** **P = 0.034**	Rho = − 0.121 P = 0.556	Rho = − 0.479 P = 0.136
I-CT	Rho = 0.299 P = 0.138	Rho = − 0.303 P = 0.132	Rho = − 0.049 P = 0.810	Rho = − 0.121 P = 0.724

Matrix correlations are presented as correlation coefficients (Rho). Abbreviations: *T1*, T category 1; *T2*, T category 2; *P*, Percentage of positive cells; *I*, Intensity; *MMP-2*, Matrix Metalloproteinase-2; *MMP-9*, Matrix Metalloproteinase-9; *TIMP-1*, Tissue Inhibitor of Matrix Metalloproteinase-1; *TIMP-2*, Tissue Inhibitor of Matrix Metalloproteinase-2; *CT*, Calcitonin in Tissue. Significance was set at *P*-values less than 0.05. Significant correlations are **boldface**

## Discussion

The present results showed no differences by MTC type for MMPs and their TIPMs, although strong TIMP-1 and TIMP-2 immunohistochemical expression of MTC was unveiled. Additionally, trace elements such as Zn, Fe, and Mn were observed to be decreased, and Cu to be increased in samples presenting MTC with respect to controls. Moreover, Zn was the unique trace element which seemed to be correlated with MMPs. These findings shed light to the idea that trace elements combined with MMPs and their TIMPs analysis could be useful markers of MTC, with Zn being the trace element of reference for this pathology.

The results of our study showed weak-moderate MMP-2, MMP-9 and strong TIMP-2, TIMP-1 immunohistochemical expression of MTC, although no differences by MTC type were observed. During cancer progression, high levels of TIMPs are associated with the inhibition of tumour growth, angiogenesis, invasion and metastasis, secondary to the inhibition of endothelial cell migration [21, 22]. Expression of MMP-9 and TIMP-2 in MTC have been analysed by other authors as well. In this regard, one study reported weak MMP and TIMP immunostaining in MTC [23]. Additionally, increased TIMP-2 levels have been suggested as a marker of low metastatic potential in MTC, since it was observed to be inversely correlated to calcitonin and tumor stage [22].

In the recent years, some studies have shown the important role of trace elements in different types of diseases including malignant tissues [24, 25] and many of the studies showed lower concentrations of Zn content in malignant tissues compared to healthy control samples [26, 27]. In line with these results, we showed a statistically significant decrease in Zn, Fe and Mn concentrations in the MTC group compared to the control group. Additionally, we demonstrated a significant increase in Cu ions concentrations in the group of samples with MTC compared to the controls. Interestingly, similar results were found in a study examining the importance of Cu concentrations in other types of thyroid carcinoma, showing a statistically significant increase in Cu concentrations of samples with thyroid carcinoma compared to the samples with Benign Thyroid Disease (BTD) [7]. A potential explanation for higher Cu concentrations in patients with thyroid carcinoma may be a possible dysregulation in metabolism of amino acids included in Cu chelating. As integral part of antioxidant enzyme Superoxide Dismutase (SOD), Zn and Cu levels could also be analysed in the context of SOD activity. Oxidative tissue damage during carcinogenesis may reduce the activity of SOD, which in turn could affect both tissue concentrations of Cu and Zn [28, 29]; however, regretfully, we did not measure SOD.

In our study, Cu, Fe, and Mn showed no relationship with MMPs, their TIMPs and CT, whereas Zn was inversely related with the MMPs of the study, but not with their TIMPs. Similarly to our results, situations of hypozincemia have been reported to be directly related to positive expression of MMPs 2 and 9, but in non-tumorous samples [30]. Moreover, although TIMPs are usually designed to interact with Zn ions in the catalytic domain, thus interfering with enzymatic activity [31], Zn did not correlate with TIMPs in our samples. Studies performed in mice, showed that in case of Zn deficiency, the expression of TIMPs was increased but the expression of MMPs was decreased [32]. Therefore, the role of Zn upon MMPs and their TIMPs may be controversial in some cases.

The present study had some limitations and strengths. As strengths, this study is one of the few of its kind published to date combining MMP-2, MMP-9 and their TIMPs in tissues with trace elements analysis. In this regard, this study emphasizes more than previous publications that assessing altogether the previously mentioned parameters could be an effective strategy for the diagnostic of MTC as useful biomarkers. As limitations, this cross-sectional study enrolled lower samples than desired. This was limited to the difficulty of finding samples presenting diagnosis of MTC who voluntary wanted to take part of the study. Moreover, the cross-sectional nature of the study was a limitation for establishing causality to the correlations observed between the main parameters of our study.

## Conclusions

The findings of the present study suggest a high tendency towards decreased levels of trace elements such as Zn, Fe, and Mn in tissues affected by MTC. In addition, Zn may be the trace element which saves more relationship with the proportion and intensity of MMPs, being altogether useful biomarkers of MTC. We therefore suggest the analysis of novel and traditional markers of MTC as a novel approach in this pathology.

## Supplementary Information

Below is the link to the electronic supplementary material.

Supplementary file1 (DOCX 50 KB)

## Data Availability

The datasets generated during and/or analysed during the current study are not publicly available due to their confidentiality but are available from the corresponding author on reasonable request.
