# Supplementary material for: Relationship between Trace Elements and Matrix Metalloproteinases 2 and 9 and their Tissue Inhibitors in Medullary Thyroid Carcinoma
Source: Biol Trace Elem Res. 2022 Sep 26;201(7):3225–32. doi: 10.1007/s12011-022-03431-z (PMC10160158; doi:10.1007/s12011-022-03431-z)
Supplement: Supplementary file 1 — Supplementary file1 (DOCX 50 KB) [file 12011_2022_3431_MOESM1_ESM.docx]

| **Supplementary Table 1.** Immunohistochemistry of calcitonin, MMPs, and TIMPs | | | | | | | |
| --- | --- | --- | --- | --- | --- | --- | --- |
| Antibody | Clone  (Cat No) | Manufacturer | Dilution | Pretreatment | Incubation with primary antibody | Visualisation kit manufacturer | Visualisation kit |
| Calcitonin | pAb  (IR515) | DAKO | RTU | water bath, pH9, 20` | 30` | Thermo Scientific™, USA | TL-125-HL |
| MMP-9 | pAb  (HPA002138) | Sigma Aldrich | 1:300 | citrate buffer, pH6 | 1h | Thermo Scientific™, USA | TL-125-HL |
| MMP-2 | pAb  (HPA001939) | Sigma Aldrich | 1:50 | microwave oven, 20`, citrate buffer, pH6 | 1h | Thermo Scientific™, USA | TL-125-HL |
| TIMP-1 | 102D1  (Ab1828) | Abcam | 1:100 | microwave oven, S1700 (Dako), 10` | 1h | Thermo Scientific™, USA | TL-125-HL |
| TIMP-2 | 3A4  (Ab1827) | Abcam | 1:100 | microwave oven, S1700 (Dako), 10` | 1h | Thermo Scientific™, USA | TL-125-HL |

| **Supplementary Table 2**. Quadrupole ICP-MS (7500ce) operating conditions | | |
| --- | --- | --- |
| **Parameters** | Characteristic | Specification |
| ***Plasma parameters*** | RF power (W) | 1500 |
|  | Plasma gas flow rate (L·min^-1^) | 15 |
|  | Auxiliary gas flow rate (L·min^-1^) | 1.18 |
| ***Reaction cell parameters*** | H_2_ gas (mL·min^-1^) | 4 |
|  | Octupole bias (V) | -18 |
|  | QP bias (V) | -16 |
| ***Data acquisition parameters*** | Monitored isotopes | Copper, Zinc, Iron, Manganese, and Gallium |
|  | Points per peak | 3 |
|  | Acquisition time per point (s) | 0.3 |
|  | Replicates | 5 |
